# Structural feature-driven pattern analysis for multitarget modulator landscapes

**DOI:** 10.1093/bioinformatics/btab832

**Published:** 2021-12-09

**Authors:** Vigneshwaran Namasivayam, Katja Stefan, Katja Silbermann, Jens Pahnke, Michael Wiese, Sven Marcel Stefan

**Affiliations:** Department of Pharmaceutical and Cellbiological Chemistry, Pharmaceutical Institute, University of Bonn, 53121 Bonn, Germany; Department of Pathology, Section of Neuropathology, Translational Neurodegeneration Research and Neuropathology Lab (www.pahnkelab.eu), University of Oslo and Oslo University Hospital, 0372 Oslo, Norway; Department of Pharmaceutical and Cellbiological Chemistry, Pharmaceutical Institute, University of Bonn, 53121 Bonn, Germany; Department of Pathology, Section of Neuropathology, Translational Neurodegeneration Research and Neuropathology Lab (www.pahnkelab.eu), University of Oslo and Oslo University Hospital, 0372 Oslo, Norway; LIED, University of Lübeck, 23538 Lübeck, Germany; Department of Pharmacology, Faculty of Medicine, University of Latvia, 1004 Rīga, Latvia; Department of Pharmaceutical and Cellbiological Chemistry, Pharmaceutical Institute, University of Bonn, 53121 Bonn, Germany; Department of Pharmaceutical and Cellbiological Chemistry, Pharmaceutical Institute, University of Bonn, 53121 Bonn, Germany; Department of Pathology, Section of Neuropathology, Translational Neurodegeneration Research and Neuropathology Lab (www.pahnkelab.eu), University of Oslo and Oslo University Hospital, 0372 Oslo, Norway; Cancer Drug Resistance and Stem Cell Program, University of Sydney, Sydney, NSW 2065, Australia

## Abstract

**Motivation:**

Multitargeting features of small molecules have been of increasing interest in recent years. Polypharmacological drugs that address several therapeutic targets may provide greater therapeutic benefits for patients. Furthermore, multitarget compounds can be used to address proteins of the same (or similar) protein families for their exploration as potential pharmacological targets. In addition, the knowledge of multitargeting features is of major importance in the drug selection process; particularly in ultra-large virtual screening procedures to gain high-quality compound collections. However, large-scale multitarget modulator landscapes are almost non-existent.

**Results:**

We implemented a specific feature-driven computer-aided pattern analysis (C@PA) to extract molecular-structural features of inhibitors of the model protein family of ATP-binding cassette (ABC) transporters. New molecular-structural features have been identified that successfully expanded the known multitarget modulator landscape of pan-ABC transporter inhibitors. The prediction capability was biologically confirmed by the successful discovery of pan-ABC transporter inhibitors with a distinct inhibitory activity profile.

**Availability and implementation:**

The multitarget dataset is available on the PANABC web page (http://www.panabc.info) and its use is free of charge.

**Supplementary information:**

Supplementary data are available at *Bioinformatics* online.

## 1 Introduction

*In silico* virtual screening approaches to identify bioactive compounds have evolved over the last few years and became fundamental for novel drug discovery. Chemical space has exponentially increased, revealing virtually endless possibilities in molecular-structural variation and composition of potentially bioactive compounds. For example, the Enamine REAL SPACE^®^ virtual compound library (https://enamine.net) consists, to this date, of over 21 billion compounds that may be obtained by organic (retro-)synthesis. Virtual screenings of this large chemical space have become not only an opportunity but also a challenge. Countless screening tools have been developed to extract putatively bioactive compounds from virtual compound libraries, but lack nowadays the necessary discriminatory potential to downsize large datasets of virtual compounds to gain high-quality compound collections.

‘Multitarget datasets’ may hold the key for sufficient discrimination of potential drug candidates. Here, the biological information of a set of known modulators of a pharmacological target is complemented with the biological information of (an)other (related or non-related) pharmacological target(s). Depending on the aimed pharmacological profile, e.g. selectivity or promiscuity, discriminators may be developed for further compound extraction. Unfortunately, such multitarget datasets are barely available (Namasivayam *et al.*, 2021a). We have recently reported a multitarget dataset of focused pan-ATP-binding cassette (ABC) transporter inhibitors (Namasivayam *et al.*, 2021c) that target the well-studied ABC transporters ABCB1, ABCC1 and ABCG2 (Namasivayam *et al.*, 2021a). These particular transport proteins constitute a model biological system for multitargeting for three reasons: (i) all three members have extensively been studied and hundreds of compounds exists that directly interact with each of the transporters; (ii) a large number of compounds exists that have been evaluated on all three targets; and (iii) a sufficient number of compounds exists with overlap in affinity regarding two or all three transporters, enabling to focus on multitargeting.

In-depth analysis of the molecular-structural patterns of the multitarget dataset resulted in the discovery of substructures with positive and negative impact on multitarget inhibition (Namasivayam *et al.*, 2021a,b). However, these extracted substructures account only for a part of the multitarget modulator landscape and do not provide sufficient discriminatory potential to downsize ultra-large virtual compound libraries.

This becomes evident considering that the Enamine drug-like^®^ virtual compound library, which was used in the aforementioned study (Namasivayam *et al.*, 2021a), revealed 1505 putatively bioactive candidates out of an initial virtual library dataset of around 16 million compounds. Comparing this initial virtual library dataset with the nowadays available ultra-large virtual library dataset of 21 billion compounds (https://enamine.net), a similar screening of the latter dataset would lead to hundreds of thousands of potential drug candidates from which manual selection is necessary. Manual selections are important to account for the empirical experience of researchers. However, mental processing of tens of thousands of compounds with consistent criteria is impossible.

In this work, we present a novel molecular-structural feature-driven pattern analysis (C@PA) workflow to explore the edges of the multitarget modulator landscape. As the prediction of highly active compounds (IC_50_ < 10 µM) in the ‘Inner Multitarget Modulator Landscape’ has already been achieved previously (Namasivayam *et al.*, 2021a), this study focused the discovery of critical substructures in the ‘Outer Multitarget Modulator Landscape’ (IC_50_ = 10–200 µM). Given the fact that a multitarget dataset of the well-studied ABC transporters ABCB1, ABCC1 and ABCG2 exists and the functional activity of these transporters can easily be assessed in well-established assays, they represent a model biological system for the intended studies. The landscape was used for the prediction of bioactive compounds with distinct bioactivity range and was biologically validated.

## 2 Materials and methods

### 2.1 Computational analyses

####  

**Compilation of dataset**. The multitarget dataset comprising 1049 manually assembled and curated compounds as reported in the literature (Namasivayam *et al.*, 2021a) has been updated by adding 111 compounds that were recently evaluated on the targets ABCB1, ABCC1 and ABCG2 (Antoni *et al.*, 2021; Namasivayam *et al.*, 2021b; Telbisz *et al.*, 2021; Vagiannis *et al.*, 2020). From this dataset of in total 1160 compounds, 138 compounds could be extracted that target all three focused targets, ABCB1, ABCC1 and ABCG2, which are termed in the following as ‘focused pan-ABC transporter inhibitors’ (Namasivayam *et al.*, 2021c), with IC_50_ values < 200 µM. In addition, a list of 304 compounds could be extracted that had either no activity (> 200 µM) or were only active against one or two transporters with IC_50_ values ≥ 10–200 µM. These compounds were termed in our previous report as ‘Class 0’ compounds (Namasivayam *et al.*, 2021a).

A scaffold search was conducted using the Structure-Activity-Report (SAReport) tool (Clark and Labute, 2009) implemented in Molecular Operating Environment (MOE; version 2019.01). In total, 103 relevant substructures were extracted from the literature (Namasivayam *et al.*, 2021a,b) as well as the present analyses. A binary distribution scheme of these substructures was established using InstantJChem (version 20.15.0). The percentage distribution of each substructure amongst the focused compounds was calculated according to formula (1).

(1) $$\%=\left(\frac{\sum \left(\mathrm{binary}\;\mathrm{values}\;\mathrm{per}\;\mathrm{substructure}\;\right)}{\sum \left(\mathrm{classified}\;\mathrm{compounds}\right)}\right)*100$$

The ratios between each group of compounds were calculated. Supplementary Table S1 provides the names and SMILES of the used compounds and substructures.

####  

**Virtual Screening**. In a recent report, we generated an ABCC1-focussing dataset comprising 1510 compounds which turned out to be an optimal virtual screening dataset due to its known multitarget hit rate of 23.5% and its non-substructure-based selection criteria (Silbermann *et al.*, 2019). It has already been established as virtual screening dataset (Namasivayam *et al.*, 2021b) and was used in this study. A virtual screening protocol was implemented applying defined conditions using InstantJChem (version 20.15.0). A manual selection of the resultant potential hit compounds provided compounds 1–5 (Supplementary Table S2) which were purchased from MolPort^®^ (http://www.molport.com).

### 2.2 Biological validation

####  

**Chemicals.** The reference ABCB1 and ABCG2 inhibitors cyclosporine A (6) and Ko143 (8) were obtained from Tocris Bioscience (Bristol, UK) and Sigma Aldrich (St. Louis, MO, USA). The reference ABCC1 inhibitor 7 was synthesized as reported earlier (Stefan *et al.*, 2017). Calcein AM and pheophorbide A were purchased from Calbiochem (EMD Chemicals, San Diego, USA). Daunorubicin has been obtained from EMD Millipore, (Billerica, MA, USA) and Hoechst 33342 was purchased from Cayman Chemicals (Ann Arbor, MI, USA). Other chemicals were purchased from Alfa Aesar (Haverhill, MA, USA), Carl Roth (Karlsruhe, Germany), Merck KgaA (Darmstadt, Germany) and VWR (Radnor, PA, USA). Compounds 1–8 were stored at -20°C as 10 mM stock solutions. Dilution series and the experimental cell culture were performed either using Krebs-HEPES buffer [KHB; 118.6 mM NaCl, 4.7 mM KCl, 1.2 mM KH_2_PO_4_, 4.2 mM NaHCO_3_, 1.3 mM CaCl_2_, 1.2 mM MgSO_4_, 11.7 mM d-glucose monohydrate and 10.0 mM HEPES (2-[4-(2-hydroxyethyl)piperazin-1-yl]ethanesulfonic acid; doubly distilled water; adjusted to pH 7.4 (NaOH); sterilized membrane filters (0.2 µm)] or colorless RPMI-1640 or Dulbecco's modified eagle media (DMEM; Sigma Life Science, Steinheim, Germany and Biowest, Nuaillé, France) cell culture media.

####  

**Cell culture.** The ABCB1-overexpressing cell line A2780/ADR and its sensitive counterpart A2780 were obtained from European Collection of Animal Cell Culture (ECACC, No. 93112520 and 93112519, respectively) and were cultivated in RPMI-1640 media supplemented with 10% fetal bovine serum (FCS) as well as streptomycin (50 µg/µl), penicillin G (50 U/ml) and l-glutamine (2 mM), all purchased from PAN-Biotech GmbH (Aidenbach, Germany) and Biowest (Nuaillé, France). The ABCC1-overexpressing cell line H69AR and its sensitive counterpart H69 were purchased from American Type Culture Collection (ATCC, No. CRL-11351 and HTB-119, respectively) and were cultivated in RPMI-1640 media supplemented with 20% FCS as well as streptomycin (50 µg/µl), penicillin G (50 U/ml) and l-glutamine (2 mM), all purchased from PAN-Biotech GmbH (Aidenbach, Germany) and Biowest (Nuaillé, France). The ABCG2-overexpressing cell line MDCK II BCRP and its wild-type MDCK II were a generous gift of Dr. A. Schinkel (The Netherlands Cancer Institute, Amsterdam, The Netherlands), and were cultivated in DMEM (Sigma Life Science, Steinheim, Germany and Biowest, Nuaillé, France) supplemented with FCS (10%), streptomycin (50 µg/µl), penicillin G (50 U/ml), as well as l-glutamine (2 mM), all purchased from PAN-Biotech GmbH (Aidenbach, Germany) and Biowest (Nuaillé, France).

All cell lines were stored in liquid nitrogen in a cell culture media/DMSO mixture (90%/10%). Cultivation was accomplished at 37°C under 5% CO_2_-humidified atmosphere. At a confluence of 90% cells were harvested using a trypsin-EDTA solution (0.05%/0.02%; PAN-Biotech GmbH, Aidenbach, Germany and Biowest, Nuaillé, France). The cells were centrifuged and the supernatant was removed, followed by resuspension of the cells in fresh cell culture media and cell counting using either a CASY TT (150 µm capillary; Schärfe System GmbH, Reutlingen, Germany) or a Scepter handheld automated cell counter (60 µM capillary sensor; EMD Millipore, Billerica, MA, USA). Subsequent seeding of cells for either sub-culturing or biological testing followed.

####  

**Calcein AM assay**. The calcein AM assay was conducted as reported earlier (Namasivayam *et al.*, 2021a,b; Silbermann *et al.*, 2019; Stefan *et al.*, 2017). For the initial screening, 20 µl of compounds 1–5 (100 µM) were added into 96-well flat-bottom clear plates (Greiner, Frickenhausen, Germany) followed by addition of 160 µl of cell suspension containing A2780/ADR (30 000 cells/well) or H69AR (60 000 cells/well) cells and incubation at 37°C and 5% CO_2_-humidified atmosphere for 30 min. Calcein AM (20 µl/3.125 µM) was added to each well and the fluorescence increase (excitation: 485 nm; emission: 520 nm) was measured in 60 sec intervals for 1 h in POLARstar and/or FLUOstar Optima microplate readers (BMG Labtech, Offenburg, Germany) and compared to the reference inhibitors 6 (ABCB1) and 7 (ABCC1). Compounds that resulted in a significantly different fluorescence increase compared with no inhibition (pure cell culture media; bottom value) were evaluated in concentration-effect curves (1–10 µM final concentration). Data processing including determination of IC_50_ values was accomplished using GraphPad Prism (version 8.4.0; San Diego, CA, USA), and the resultant concentration-effect curves have been constrained to the maximal effect of standard inhibitors 6 (ABCB1) and 7 (ABCC1).

####  

**Pheophorbide A assay**. The pheophorbide A assay was conducted as reported earlier (Namasivayam *et al.*, 2021a,b; Silbermann *et al.*, 2019). Compounds 1–5 (100 µM) were added into 96-well flat-bottom clear plates followed by addition of 160 µl of cell suspension containing MDCK II BCRP (45 000 cells/well) and 20 µl of a pheophorbide A solution (5 µM) with subsequent incubation at 37°C and 5% CO_2_-humidified atmosphere for 120 min. Flow cytometry (Guava easyCyte^TM^ HT; Merck Millipore, Billerica, MA, USA; excitation: 488 nm; emission: 695/50 nm) was applied, and the average fluorescence value per cell per well was correlated to the used compound concentration of compounds 1–5 as well as reference inhibitor 8. Compounds that resulted in a significantly different fluorescence increase compared to no inhibition (pure cell culture media; bottom value) were evaluated in concentration-effect curves (1–10 µM final concentration). Data processing was conducted as stated above, and the resultant concentration-effect curves have been constrained to the maximal effect of standard inhibitor 8.

####  

**Daunorubicin assay**. For the verification of determined IC_50_ values against ABCB1 and ABCC1 in the calcein AM assay and to exclude unspecific effects, the daunorubicin accumulation assay was performed as described earlier (Jekerle *et al.*, 2006; Namasivayam *et al.*, 2021a). Dilution series of hit compounds 3 and 5 between concentrations of 5–300 µM have been generated and 20 μl of the compounds were added into clear 96-well flat-bottom plates (Greiner, Frickenhausen, Germany) and complemented with 160 μl of a cell suspension containing 45 000 cells of either A2780/ADR, A2780, H69AR or H69 cell lines in colorless RPMI-1640 without supplements. Twenty microliters of a daunorubicin solution (30 µM) were added and the plate was incubated for 180 min at 37°C and a 5% CO_2_ humidified atmosphere. Fluorescence was measured with an Attune NxT flow cytometer (Invitrogen, Waltham, MA, USA) at an excitation wavelength of 488 nm and an emission wavelength of 695/50 nm. Data analysis was performed as described before, and the resultant concentration-effect curves have been constrained to the maximal effect of standard inhibitors 6 (ABCB1) and 7 (ABCC1).

####  

**Hoechst 33342 assay.** To verify the inhibition against ABCG2 and to exclude unspecific effects of compounds 3 and 5 obtained in flow cytometry, we performed a plate reader-based Hoechst 33342 assay as reported earlier (Namasivayam *et al.*, 2021a). Dilution series of test compounds 3 and 5 between concentrations of 5–300 µM have been generated and 20 μl of the test compounds were added into black 96-well flat-bottom plates (Brand, Wertheim, Germany) and complemented with 160 μl of a cell suspension containing 30 000 cells of either MDCK II BCRP or MDCK II cells in colorless DMEM without supplements. After an incubation period of 30 min at 37°C and 5% CO_2_, 20 µl of a Hoechst 33342 solution (10 μM) were added. The fluorescence accumulation at an excitation wavelength of 360 nm and an emission wavelength of 460 nm was measured over a period of 120 min in 60 s intervals using a Paradigm microplate reader [Beckman Coulter (Brea, CA, USA)]. The average steady-state fluorescence value was calculated for each well and correlated to the used compound concentration. Data analysis was performed as described before, and the resultant concentration-effect curves have been constrained to the maximal effect of standard inhibitor 8.

####  

**Fluorimetric analyses.** Compounds 3 and 5 (5–30 µM final concentration) were subject to fluorescence measurements for the determination of potential auto-fluorescence. Excitation wave-lengths have been analyzed that were used in the described assays above [360 (Hoechst 33342) and 485 (calcein AM, daunoruicin, pheophorbide A)] by acquiring complete spectra covering a wavelength range between 400–700 and 525–700 nm, respectively, using a Paradigm microplate reader [Beckman Coulter (Brea, CA, USA)]. Neither compound 3 nor compound 5 showed auto-fluorescence at concentrations up to 30 µM (data not shown).

## 3 Results

### 3.1 Classification of compounds

**Fig. 1.**
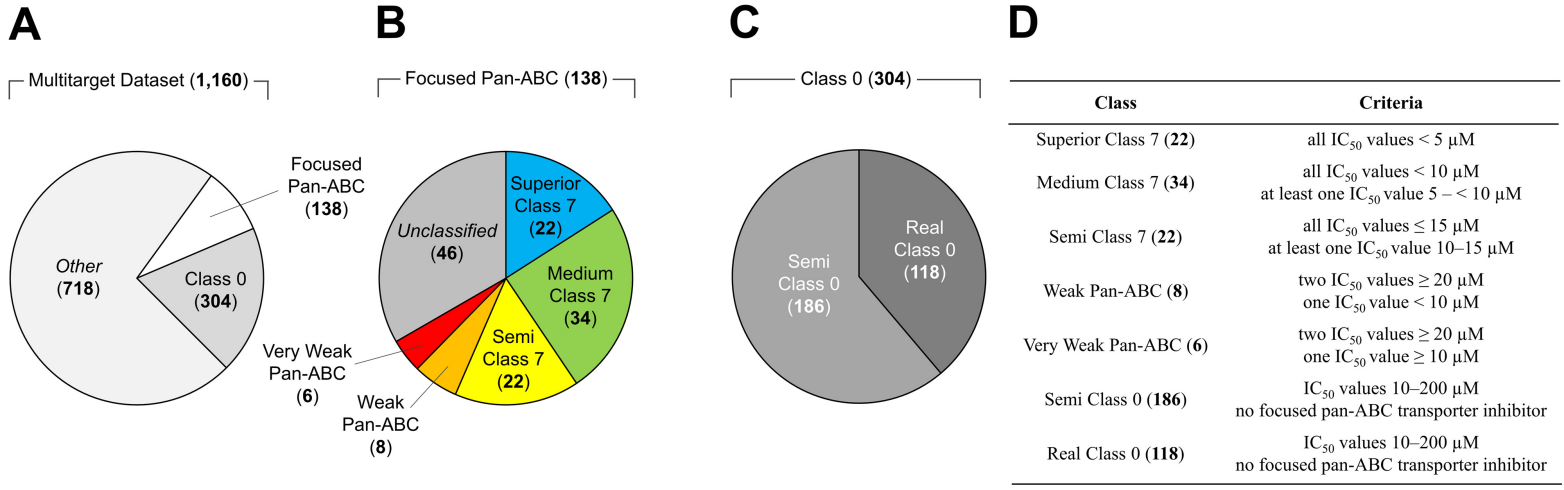
Pie diagrams of compound classes as defined within our previous (Namasivayam *et al.*, 2021a,b,c) and the present work. (**A**) The entire multitarget dataset and within this study used 138 focused pan-ABC transporter inhibitors as well as inactive Class 0 compounds. (**B**) The sub-classification of focused pan-ABC transporter inhibitors into unclassified compounds and classified focused pan-ABC transporter inhibitors. (**C**) The sub-classification of class 0 compounds. (**D**) Criteria for sub-classification of classified focused pan-ABC transporter inhibitors and class 0 compounds

As specified in Section 2.1, 138 focused pan-ABC transporter inhibitors were extracted from the multitarget dataset (Antoni *et al.*, 2021; Namasivayam *et al.*, 2021a,b; Telbisz *et al.*, 2021; Vagiannis *et al.*, 2020; Fig.  1A). These compounds were classified according to their activity profile (from very strong to very weak inhibition; Fig.  1B). In our earlier report, compounds with IC_50_ values < 10 µM were designated as ‘Class 7’ compounds (Namasivayam *et al.*, 2021a). In the 138 focused pan-ABC transporter inhibitors, 56 Class 7 compounds exist. However, to acknowledge the different inhibition values and to allow for a detailed analysis, we sub-categorized these compounds into ‘*Superior Class 7*’ (IC_50_ values against all targets < 5 µM; 22 of 138 compounds) and ‘*Medium Class 7*’ compounds (all IC_50_ values < 10 µM and at least one IC_50_ value between 5 and < 10 µM; 36 of 138 compounds). In addition, we established ‘*Semi Class 7*’ compounds in our previous work that had significant potency against all three selected targets, ABCB1, ABCC1 and ABCG2, however, did not qualify for class 7 compounds (Namasivayam *et al.*, 2021b): all IC_50_ values ≤ 15 µM and at least one IC_50_ value 10–15 µM; 22 of 138 compounds. The compound classification was completed by defining weak and very weak focused pan-ABC transporter inhibitors. ‘*Weak Pan-ABC*’ transporter inhibitors were defined by IC_50_ values against two transporters ≥ 20 µM and against one transporter < 10 µM, which accounted for 8 of 138 compounds). ‘*Very Weak Pan-ABC*’ transporter inhibitors were defined by IC_50_ values against two transporters ≥ 20 µM and against one transporter ≥ 10 µM; 6 of 138 compounds). In summary, 92 (22 + 34 + 22 + 8 + 6) of the 138 focused pan-ABC transporter inhibitors could be categorized according to the described scheme, which will be termed in the following as ‘classified focused pan-ABC transporter inhibitors’. On the other hand, 46 of 138 compounds could not be taken into account in the following analyses and were termed as ‘unclassified’.

In addition to the collection of pan-ABC transporter inhibitors, 304 compounds could be extracted from the multitarget dataset that had either (almost) no activity toward the transporters ABCB1, ABCC1 and ABCG2 (IC_50_ > 200 µM; 118 of 304 compounds) or affected maximally 2 transporters with activity values between 10–200 µM (186 of 304 compounds; Antoni *et al.*, 2021; Namasivayam *et al.*, 2021b; Telbisz *et al.*, 2021; Vagiannis *et al.*, 2020; Fig.  1C). These compounds are referred to in the following as ‘Class 0’ compounds according to our previous report (Namasivayam *et al.*, 2021a). However, to enable for a detailed analysis of this particular set of compounds, the 118 compounds without any activity were designated as ‘*Real Class 0*’ compounds, while the 186 compounds were named ‘*Semi Class 0*’ compounds. Figure  1D summarizes the criteria of compound classification for subsequent statistical pattern analysis.

### 3.2 Compilation of relevant substructures

Our previous reports have revealed several critical substructures in terms of multitarget inhibition of the transporters ABCB1, ABCC1 and ABCG2, which can generally be distributed into two different groups: (i) the ‘positive multitarget substructures’ that promote pan-ABC transporter inhibition; and (ii) the ‘negative multitarget substructures’ which impedes pan-ABC transporter inhibition (Namasivayam *et al.*, 2021a,b). Taking our previous literature reports into account, the positive multitarget substructures consist of (i) 6 ‘primary basic scaffolds’, (ii) 2 ‘suggested basic scaffolds’, (iii) 7 ‘primary positive substructures’; (iv) 7 ‘suggested positive substructures’, (vi) 15 ‘extended positive substructures’ and (vii) 9 ‘putative positive substructures’. On the other hand, the negative multitarget substructures comprise 30 ‘primary negative substructures’. A detailed survey of these substructures and their allocated group can be found in Supplementary Table S3.

### 3.3 Extension of multitarget substructures

As stated above, 6 primary basic scaffolds of pan-ABC transporter inhibitors were identified [(i) 4-anilinopyrimidine; (ii) pyrrolo[3,2-*d*]pyrimidine; (iii) pyrimido[5,4-*b*]indole; (iv) quinazoline; (v) quinoline; (vi) thieno[2,3-*b*]pyridine; Namasivayam *et al.*, 2021a]. However, these primary basic scaffolds were obtained based on the 48 compounds identified as Class 7 compounds at the time of the study—and this number has now increased to 56 compounds (Antoni *et al.*, 2021; Namasivayam *et al.*, 2021a,b; Riganti *et al.*, 2019; Sachs *et al.*, 2019; Telbisz *et al.*, 2021). In addition, the molecular-structural information of focused pan-ABC transporter inhibitors of ABCB1, ABCC1 and ABCG2 with activities ≥ 10 µM had been ignored in our previous study. Nevertheless, this molecular-structural information is particularly important to uncover the entire multitarget modulator landscape. Hence, we conducted again a basic scaffold search with the 138 known focused pan-ABC transporter inhibitors applying SAReport and identified 3 ‘extended basic scaffolds’ [(i) tetrahydroisoquinoline; (ii) benzochromenone and (iii) chromenone] which complement the positive multitarget substructures (Supplementary Table S3).

In our most recent work, we suggested putative positive substructures that were defined as partial structures which were present in at least 10 Class 7 compounds (Namasivayam *et al.*, 2021b). However, no analogous concept has been developed for negative multitarget substructures. Hence, we re-analyzed the multitarget dataset (Namasivayam *et al.*, 2021a) toward ‘putative negative substructures’, and identified 24 additional substructures that complement the negative multitarget substructures according to a rule scheme as outlined in Supplementary Information S1.

In total, 103 substructures have been compiled from the literature (Namasivayam *et al.*, 2021a,b) and this study (Supplementary Table S3), which were used for subsequent analyses.

### 3.4 Individual pattern analysis and substructural re-grouping

To obtain a detailed understanding of the individual contribution of each of the 103 substructures to multitarget inhibition of the ABC transporters ABCB1, ABCC1 and ABCG2 and to identify important substructures in terms of the multitarget modulator landscape, an individual evaluation of the statistical distribution of these 103 substructures amongst the 92 classified focused pan-ABC transporter inhibitors of ABCB1, ABCC1 and ABCG2, as well as the 304 Class 0 compounds for each substructure has been conducted. This ‘individual pattern analysis’ followed a rule scheme as outlined in Supplementary Information S2, and the individual substructures were evaluated and re-grouped according to a point score (+++; ++; +; 0) depending on the (non-)violation of the rules as outlined in Supplementary Information S3.

Considering the point scores, the following groups were defined in which the 103 analyzed substructures could be re-distributed: (i) ‘*Superior Inner Multitarget Modulator Landscape*’ [strong contribution (+++) in terms of potent (<10 µM) pan-ABC transporter inhibition; 6 of 103 substructures], (ii) ‘*Inferior Inner Multitarget Modulator Landscape*’ [moderate contribution (++) in terms of potent (<10 µM) pan-ABC transporter inhibition; 13 of 103 substructures], (iii) ‘*Superior Outer Multitarget Modulator Landscape*’ [strong contribution (+++) in terms of weak (10–200 µM) pan-ABC transporter inhibition; 14 of 103 substructures], (iv) ‘*Inferior Outer Multitarget Modulator Landscape*’ [moderate contribution (++) in terms of weak (10–200 µM) pan-ABC transporter inhibition; 5 of 103 substructures] and (v) ‘*Intermediate Substructures*’ [no clear contribution to either landscape (+/+; +/0; 0/+; 0/0); in total 65 of 103 substructures]. It should be noted that ‘amino’ and ‘indole’ have been equally evaluated in terms of the Inner and Outer Multitarget Modulator Landscape (++) and were allocated to the Inferior Inner Multitarget Modulator Landscape.

A closer look at the statistical distribution of the 65 Intermediate Substructures amongst all 138 focused pan-ABC transporter inhibitors revealed critical sub-populations of substructures: (v-i) ‘*Inconclusive Substructures*’ (in total 6 of 103 substructures) that either appeared amongst all groups with an equal distribution (3 of 103 substructures) or have not been found in any group (3 of 103 substructures); (v-ii) ‘*Tolerated Negative Substructures*’ which were found in Semi Class 0 compounds that had weak/very weak (10–200 µM) inhibition of one or two targets and, in addition, were not found in the unclassified compounds (17 of 103 substructures) and (v-iii) ‘*Untolerated Negative Substructures*’ that occurred only in Real Class 0 compounds, and hence, impeded pan-ABC transporter inhibition (2 of 103 substructures). Except for the Tolerated Negative Substructures and Untolerated Negative Substructures, Intermediate Substructures were omitted in the following computing steps to widen the gap between the Inner and Outer Multitarget Modulator Landscape.

**Fig. 2.**
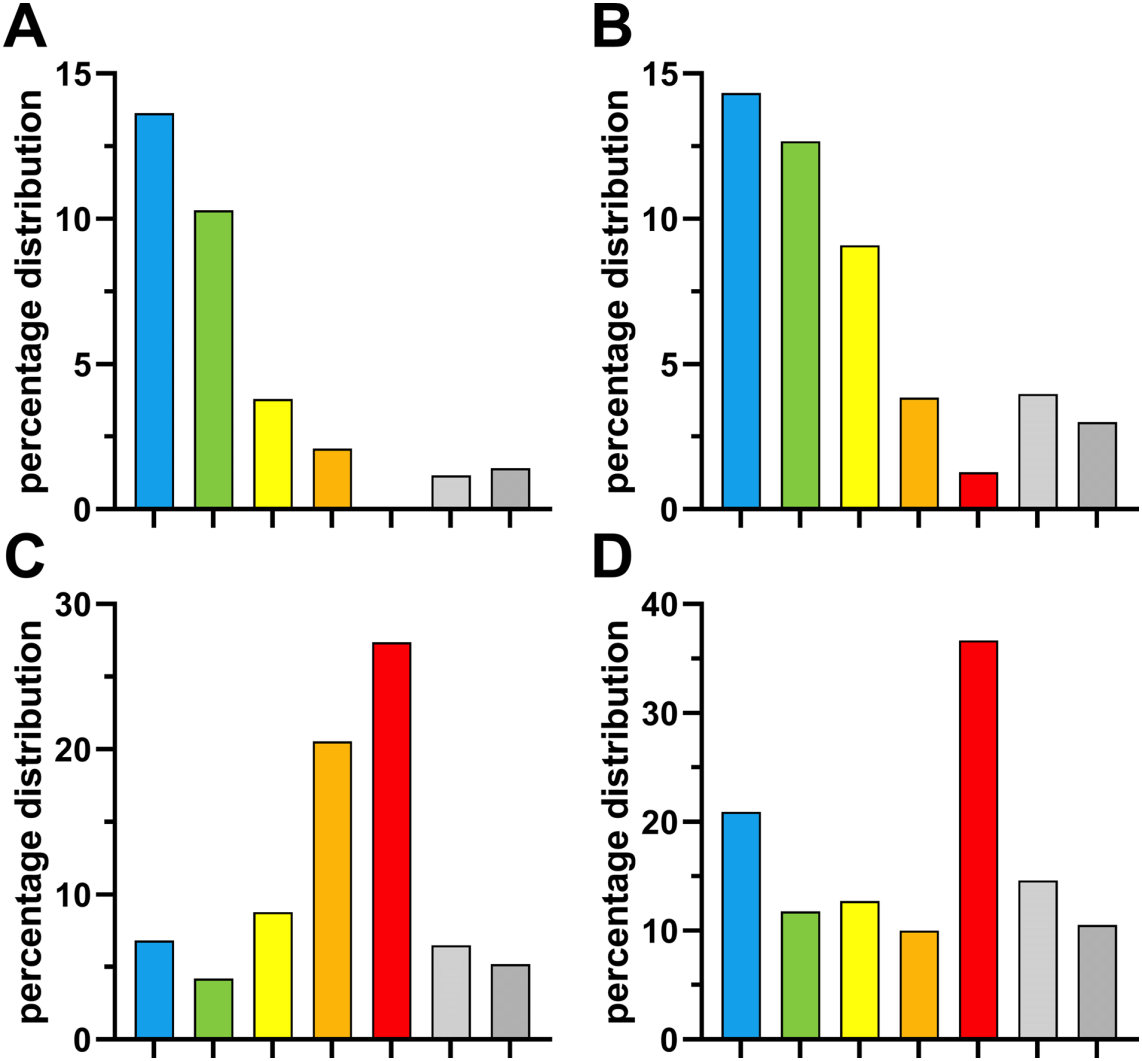
Percentage distribution of the 38 (of 103) relevant positive and negative multitarget substructures amongst the 92 (of 138) classified grouped focused pan-ABC transporter inhibitors as well as the 304 Class 0 compounds. (**A**) Superior Inner Multitarget Modulator Landscape substructures (6 of 103 substructures). (**B**) Inferior Inner Multitarget Modulator Landscape substructures (13 of 103 substructures). (**C**) Superior Outer Multitarget Modulator Landscape substructures (14 of 103 substructures). (**D**) Inferior Outer Multitarget Modulator Landscape substructures (5 of 103 substructures). Color coding: blue = Superior Class 7; green = Medium Class 7; yellow = Semi Class 7; orange = Weak Pan-ABC transporter inhibitors; red = Very Weak Pan-ABC transporter inhibitors; gray = Semi Class 0 compounds; anthracite = Real Class 0 compounds

Supplementary Table S3 visualizes the relevant substructures in terms of the Inner and Outer Multitarget Modulator Landscape, as well as their point score. Figure  2 provides the statistical distribution of the substructures of the Superior Inner (Fig.  2A), Inferior Inner (Fig.  2B), Superior Outer (Fig.  2C) and Inferior Outer (Fig.  2D) Multitarget Modulator Landscape amongst the 92 classified focused pan-ABC transporter inhibitors as well as the 304 Class 0 compounds.

### 3.5 Virtual screening for the outer multitarget modulator landscape

To support the presented discoveries, we implemented a virtual screening protocol specifically to discover compounds of the Outer Multitarget Modulator Landscape. For this purpose, the already established virtual screening dataset of 1510 compounds was applied as described before (Namasivayam *et al.*, 2021b; Silbermann *et al.*, 2019). This particular dataset has a known multitarget hit rate of 23.5% and was not constrained to C@PA-derived inclusion- and/or exclusion criteria.

**Fig. 3.**
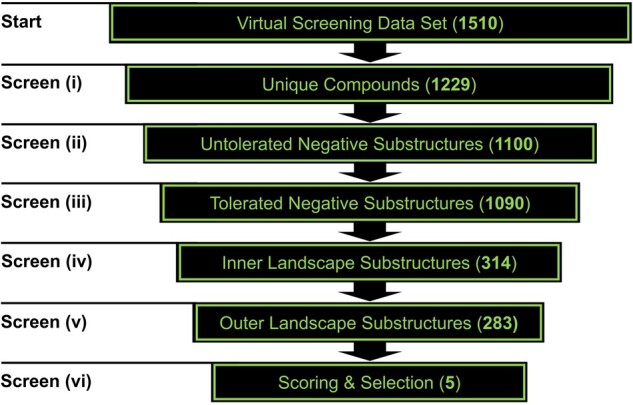
Virtual screening workflow presented in this work for the exploration of the Outer Multitarget Modulator Landscape

**Fig. 4.**
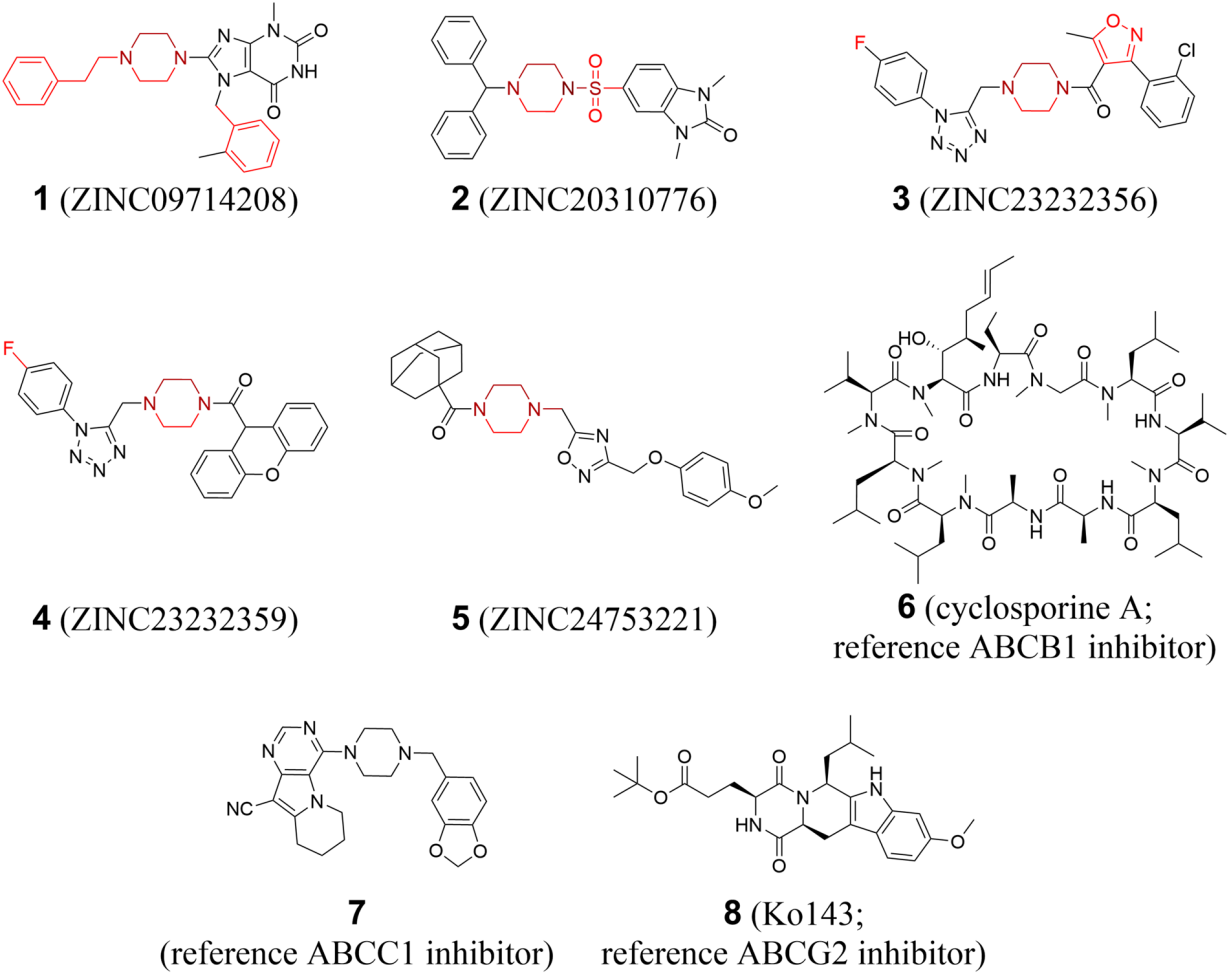
Hit compounds 1–5 obtained from the Outer Multitarget Modulator Landscape-focused virtual screening approach of this work. Cyclosporine A (6), compound 7 and Ko143 (8) were used as ABCB1, ABCC1 and ABCG2 reference inhibitors, respectively. Focused Outer Multitarget Modulator Landscape substructures are highlighted in red; overlapping/concentrated sbstructures are highlighted in different red tones

The 1510 compounds were subjected to 6 screening steps: (i) removal of redundant compounds (stereoisomers) to increase the diversity of the compound set as demonstrated earlier (Namasivayam *et al.*, 2021b; ‘Unique Compounds'; 1229 in/281 out); (ii) removal of Untolerated Negative Substructures (‘Untolerated Negative Substructures'; 1100 in/129 out); (iii) removal of compounds with more than one Tolerated Negative Substructure (‘Tolerated Negative Substructures'; 1090 in/10 out); (iv) removal of compounds with Inner Multitarget Modulator Landscape substructures (‘Inner Landscape Substructures'; 314 in/776 out); (v) removal of compounds that contained no Outer Multitarget Modulator Landscape substructure (‘Outer Landscape Substructure'; 283 in/31 out) and (vi) scoring of resultant compounds and procurement for biological evaluation (5 in/278 out). In summary, 30, 76, 102, 53, 19 and 3 compounds contained 1, 2, 3, 4, 5 and 6 Outer Multitarget Modulator Landscape substructures, respectively. Compounds 1–5 (Supplementary Table S2) were manually selected and purchased from MolPort^®^ (http://www.molport.com) depending on the number (1–3: 4; 4: 3; 5: 2), manner (1: benzyl, ethylenediamine, phenethyl and piperazine; 2: ethylenediamine, piperazine, sulfone, thioether; 3: ethylenediamine, fluorine, isoxazole and piperazine; 4: ethylenediamine, fluorine and piperazine and 5: ethylenediamine and piperazine), as well as composition (1: distributed; 2: concentrated; 3: distributed; 4: distributed; 5: concentrated) of Outer Multitarget Modulator Landscape substructures. To ensure a high molecular-structural diversity amongst the compounds, basic scaffolds were chosen different from primary or extended basic scaffolds as mentioned above (1: xanthine; 2: benzimidazolidine; 3: isoxazole and tetrazole; 4: tetrazole and xanthene 5: 1,2,4-oxadiazole). Additionally, commercial availability (e.g. producibility or purity) and affordability (e.g. price or time to synthesize/deliver) were additional selection criteria. Unfortunately, none of the 22 compounds containing either 5 or 6 Outer Multitarget Modulator Landscape substructures could be obtained. The exact screening substructures are outlined in Supplementary Table S4. Figure  3 provides the used virtual screening workflow, while Figure  4 shows compounds 1–5 which were subject to biological evaluations.

### 3.6 Biological validation of the outer multitarget modulator landscape

**Fig. 5.**
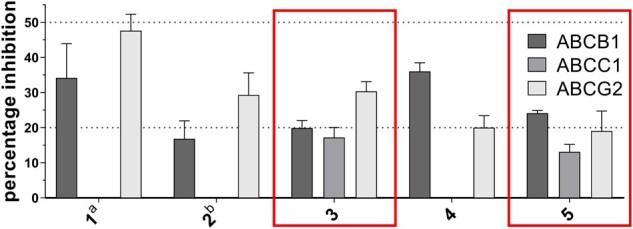
Investigation of compounds 1–5 (10 µM) against ABCB1 and ABCC1 (calcein AM assay), and ABCG2 (pheophorbide A assay). Normalization was conducted by defining the effect of 10 µM of compounds 6 (ABCB1), 7 (ABCC1) and 8 (ABCG2) as 100% and pure cell culture medium as 0%. The mean values ± standard error of the mean (SEM) of at least three independent experiments are shown. Red mark: discovered Outer Multitarget Modulator Landscape-focused pan-ABC transporter inhibitors 3 and 5; ^a^apparent ABCC1 activation (5.7% ± 2.7%); ^b^apparent ABCC1 activation (7.0% ± 2.8%)

Calcein AM (ABCB1 and ABCC1) and pheophorbide A (ABCG2) fluorescence accumulation assays were used to evaluate the inhibitory feature of compounds 1–5. For this purpose, ABCB1-overexpressing A2780/ADR, ABCC1-overexpressing H69AR, and ABCG2-overexpressing MDCK II BCRP were used as reported earlier (Namasivayam *et al.*, 2021a,b; Silbermann *et al.*, 2019; Stefan *et al.*, 2017). Figure  5 shows the results of the initial screening of the compounds at 10 µM, and Table  1 reveals the inhibitory activity data of the compounds that resulted in a significant fluorescence increase compared to no inhibition (pure cell culture media; bottom value). The obtained IC_50_ values have been compared to very weak pan-ABC transporter inhibitors known in the literature (6 compounds; Colabufo *et al.*, 2009; Lempers *et al.*, 2016; Obreque-Balboa *et al.*, 2016).

**Table 1.** Inhibitory activity of compounds 1–5 against ABCB1, ABCC1 and ABCG2 expressed as IC_50_ values derived from full-down concentration–effect curves determined in plate reader-based calcein AM (ABCB1 and ABCC1) and Hoechst 33342 (ABCG2) assays, as well as flow cytometer-based daunorubicin (ABCB1 and ABCC1) and pheophorbide A (ABCG2) assays as reported earlier (Jekerle *et al.*, 2006; Namasivayam *et al.*, 2021a,b; Sibermann *et al.*, 2019; Stefan *et al.*, 2017) compared to IC_50_ values of very weak pan-ABC transporter inhibitors as reported earlier (Colabufo *et al.*, 2009; Lempers *et al.*, 2016; Obreque-Balboa *et al.*, 2016)

Compound	IC_50_±SEM (μM)	IC_50_±SEM (μM)	IC_50_±SEM (μM)	IC_50_±SEM (μM)	IC_50_±SEM (μM)	IC_50_±SEM (μM)
	ABCB1 Calcein AM	ABCB1 Daunorubicin	ABCC1 Calcein AM	ABCC1 Daunorubicin	ABCG2 Pheophorbide A	ABCG2 Hoechst 33342
1	22.7±5.4	n.t.	n.i.	n.t.	7.89±0.44	n.t.
2	8.26±0.54	n.t.	n.i.	n.t.	25.8±12.1	n.t.
3	33.1±3.7	80.5±19.4	38.6±2.2	38.7±15.2	15.9±1.1	37.9±12.5
4	15.5±1.3	n.t.	n.i.	n.t.	15.1±1.2	n.t.
5	25.6±1.1	80.2±30.1	60.9±14.0	57.0±6.5	12.4±1.3	28.5±6.1
Very weak pan-ABC transporter inhibitors	IC_50_±SEM (μM) ABCB1 Literature		IC_50_±SEM (μM) ABCC1 Literature		IC_50_±SEM (μM) ABCG2 Literature	
2-Aryloxazole derivative 8f^a^	32.8±3.2		70±2.5		15±1.2	
2-Aryloxazole derivative 8h^a^	12.7±4.0		86±6.5		90±4.2	
Flavonoid derivative 33^b^	33±4.6		10.9±1.8		36.9±6.7	
Flavonoid derivative 35^b^	58.5±6.0		38.0±16.0		66.6±11.3	
Flavonoid derivative 39^b^	32.8±3.9		18.8±7.6		38.2±6.4	
Micafungin^c^	45 (29.2–70.5)		21 (19.9–23.0)		21 (17.4–25.4)	

*Note*: The top values of the measured concentration–effect curves have been constrained to the top value of the reference compounds 6 (ABCB1), 7 (ABCC1) and 8 (ABCG2). n.i., no inhibition; n.t., not tested; ^a^Reported earlier (Colabufo *et al.*, 2009); ^b^Reported earlier (Obreque-Balboa *et al.*, 2016); ^c^Reported earlier (Lempers *et al.*, 2016).

Compounds 1–5 had at least inhibitory activities against two targets, and hence, revealed themselves as multitarget inhibitors. This equals a multitarget hit rate of 100%. This multitarget hit rate exceeded the determined multitarget hit rates of our previous studies [70.0% (Namasivayam *et al.*, 2021b) and 60.9% (Namasivayam *et al.*, 2021a)]. Two candidates, 3 and 5, targeted ABCB1, ABCC1 and ABCG2, which represents a hit rate of 40%. This hit rate is comparable to the hit rate of our previous study (Namasivayam *et al.*, 2021b), and higher than similar virtual screening approaches [21.7% (Namasivayam *et al.*, 2021a) and 23.5% (Silbermann *et al.*, 2019)]. The standout findings are the activity ranges of these compounds. As intended, compounds 3 and 5 were identified as pan-ABC transporter inhibitors of the Outer Multitarget Modulator Landscape. In addition, the measure to include one tolerated negative multitarget substructure to acknowledge certain bioactivities of Semi Class 0 compounds proved to be correct. Tetrazole, a substructure mostly occurring in real class 0 compounds and rarely present in Semi Class 0 compounds, can now be considered as an ‘active substructure' in terms of multitargeting of ABC transporters.

To confirm our results and to exclude unspecific effects or assay-related artifacts in terms of the hit compounds 3 and 5, we determined inhibition values in alternative assays. For this purpose, the flow cytometry-based dauorubicin accumulation assay (ABCB1 and ABCC1) as well as the plate reader-based Hoechst 33342 assay have been chosen. In addition, we investigated compounds 3 and 5 at various concentrations in sensitive/wild-type cell lines (A2780, H69 and MDCK II). In essence, the inhibitory power against the targets ABCB1, ABCC1 and ABCG2 could be confirmed in all three cases (Table  1), and no unspecific effects were identified after taking the data regarding the sensitive/wild-type cell lines into account. Supplementary Figure S1 demonstrates the concentration-effect curves generated for the most potent representative of the newly derived very weak pan-ABC transporter inhibitors, compound 5.

## 4 Conclusions

At first glance, the intended search and prediction of weak multitargeting compounds seems counterintuitive. In the past, virtual screening approaches focused on the identification of potential drug candidates with high potency toward their target(s) (Namasivayam *et al.*, 2021a,b; Silbermann *et al.*, 2019). However, the goal of this study was to address the question of whether additional descriptional substructures could be identified and/or subsequently developed that define the multitarget modulator landscape in its entirety, and may be used to search for a specific activity range of bioactive compounds. The multitarget modulator landscape is particularly of major interest due to five reasons.

First, with the advance of chemical space, new descriptors are needed to downsize ultra-large virtual compound libraries. Conventional screening methodologies with rather simple selection frameworks will not be sufficient in the future to efficiently reduce the absolute number of potential drug candidates. Indeed, countless screening tools have been identified in the last 2–3 decades, and the number of descriptors is almost endless. However, certain descriptors used as additional discriminators may not be directly connected to bioactivity and could lead to false selections or even to no discrimination in terms of the outgoing virtual screening dataset. This could lead to an insufficient reduction of potential drug candidates, and hence, an immense output of compounds that need to be processed manually. As outlined in the introduction, manual selections support the drug selection process through empirical experience of researchers. However, this process should be reserved for the final shortlisted compounds, not tens of thousands of compounds, to keep selection criteria consistent and prevent irrational or random selection. Rationalizing these decisions to the very end is the major obstacle in computational chemistry today.

In terms of the selection process, the Outer Multitarget Modulator Landscape in general can serve in two ways: (i) as an additional descriptor to target a specific bioactivity range and/or to support the extraction of the potentially best compounds for *in vitro* and subsequently *in vivo* experiments, as well as (pre-)clinical evaluation of a specific virtual screening approach; and (ii) to create high-quality compound collections that serve multifactorial purposes. These compound collections are pre-selected compound sets with target-related determinants from which an actual screening for bioactivity takes place, for example, to establish new screening methodologies.

Very recently, C@PA was introduced as a novel screening methodology ultimately identifying substructures of compounds which were linked to bioactivity, and developing these substructures as descriptors for virtual screening approaches (Namasivayam *et al.*, 2021a). Their subsequent extension proved how powerful substructures really are (Namasivayam *et al.*, 2021b). Through the presented workflow, several substructures could be identified that outline and mark the very edges of the Outer Multitarget Modulator Landscape, which qualify as additional descriptors for pre-selecting ultra-large virtual screening compound libraries for the generation of high-quality compound collections, and ultimately the final rational drug selection process. In addition, the presented individual statistical pattern analysis will promote fragment-based *de novo* computational drug design approaches.

Second, the interest in polypharmacology has risen in recent years (Bajorath, 2021; Blaschke and Bajorath, 2021; Feldmann and Bajorath, 2020; Stefan, 2019; Stefan *et al.*, 2020a). The presented methodology proved to be superior in extracting multitargeting compounds, which may be transferred to other fields of pharmacology as well. However, another reason to target the Outer Multitarget Modulator Landscape could be the development of novel agents of targets that are under-studied and cannot be addressed yet by small molecules. These small molecules may be promising for the exploration and exploitation of novel pharmacological targets and the development of new diagnostics and therapeutics against human diseases. With the knowledge of the Outer Multitarget Modulator Landscape, new template structures can be derived, which may subsequently be improved by derivatization, initiating rational drug design. In the case of the model biological system of ABC transporters, this multitarget exploration concept has very recently been introduced, and (a) similar or at least overlapping common binding site(s) of pan-ABC transporter inhibitors across different ABC transporter subfamilies has been proposed (Namasivayam *et al.*, 2021a,b,c; Stefan *et al.*, 2020a). This ‘multitarget binding site’ may hold the key to address under-studied ABC transporters and target under-studied ABC transporters-associated diseases.

Third, drug development does not only focus on polypharmacology but also on selective compounds. However, to generate purely selective compounds, the knowledge of both the inner and outer bioactivity landscape of these compounds toward several (related or unrelated) targets is critical. The presented workflow allows for the identification of these critical substructures to design compounds with the intended pharmacological profile. The outer bioactivity landscape is particularly of interest to avoid potential side-effects in (pre-)clinical evaluations. In terms of interference with ABC transporters, this extended pharmacological profile is of specific importance. ABC transporters are mainly expressed in blood-tissue barriers, and hence, majorly influence the distribution, pharmacokinetic, and therefore, pharmacodynamic of drugs. The design of, or screening for, drug candidates that do not bear features of the Inner and Outer Multitarget Modulator Landscape of pan-ABC transporter inhibition is highly preferable in the drug design processes.

Fourth, computational chemistry approaches with respect to the exploration of the bioactivity landscapes of compound datasets support the exploration of the bioactivity space in the vastly growing chemical space. The great advantage of virtual screening approaches is the almost certain discovery of novel compounds with unique molecular-structural composition and orientation. This also leads to the discovery of novel mode of actions. For example, one compound of our previous study (Namasivayam *et al.*, 2021b) and two of this study (compounds 1–2) revealed an apparent slight activation of ABCC1. Activators of this ABC transporter are indeed known (Stefan and Wiese, 2019; Wiese and Stefan, 2019), however, only very few compounds had distinct molecular features apart from known intrinsic ABCC1 substrates. The discovery and exploration of novel substructures associated with different modes of modulation will allow for the exploration of multiple dimensions of bioactivity space, for example, functional (mode of modulation) and structural (mode of binding) aspects. In terms of ABC transporters, the active/intended search of ABC transporter activators is a current research field that would benefit from these developments.

Fifth, feature-driven C@PA (‘C@PA_1.3’) offers the opportunity to establish certain substructures with distinct pharmacological profile into a collective set of substructures for subsequent screening of virtual compound libraries. This may be of importance, for example, if different substructure sets targeting different (related or unrelated) targets, shall be combined to pursue a polypharmacological approach. Here, a ‘collective pattern analysis’ combining two different sets of substructures may reveal the positive or negative impact of (a) certain introduced substructure(s) on the bioactivity range of a training dataset. Also, the introduction of new or negative substructures (e.g. tetrazole) that are not associated with (a) certain target(s) may be accomplished with collective pattern analysis. This in effect shapes not only the future substructure composition in virtual screening approaches, but also the extracted compounds derived from these virtual screening approaches. These measures promote a steady re-consideration and re-evaluation of existing datasets, thriving novel and innovative drug development.

The virtual screening workflow of the future is suggested to comprise (i) an enlarged substructure dataset. The more substructures are searched for, the more discriminatory potential exists to downsize ultra-large virtual screening compound libraries to generate high-quality compound collections and to understand bioactivity landscapes as a whole; (ii) statistical pattern analysis according to the original C@PA (Namasivayam *et al.*, 2021a), which proved to give an important framework for the ongoing substructural evaluation; (iii) scaffold fragmentation and substructure hopping with positive and negative multitarget substructures. The extension of the positive and negative multitarget substructures proved to be a significant discovery in terms of the exploration of pattern analysis in our previous (Namasivayam *et al.*, 2021b) and this work; (iv) individual and collective pattern analysis of featured substructures. Specific substructures will be of higher importance than others. The current work presents a detailed scheme on how these important substructures may be elucidated and harnessed for better discriminatory potential; and (v) adjustment of virtual screening parameters and subsequent virtual screening. In the final step, gained knowledge from steps (i) to (iv) must lead to a synthesized discrimination scheme that sufficiently downsized ultra-large virtual screening compound libraries.

Feature-driven pattern analysis for the elucidation of the multitarget modulator landscapes can and will be used for other (human or non-human) pharmacological targets as well, for example, other membrane bound proteins [e.g. under-studied human/bacterial ABC transporters, G-protein coupled receptors (GPCRs), ion channels (ICs), solute carriers (SLCs; PANSLC) or tyrosine kinases (TKs)]. The landscape is not only bound to the biological activity (e.g. inhibition). Other biological properties may also be subject to this methodology, for example, the mode of action (e.g. activation or partial inhibition), the binding(-site) behavior (e.g. competitive, non-competitive, or uncompetitive), the target localization/drug effect venue [e.g. cell membrane or intra-vesicular systems (Stefan *et al.*, 2020a,b)] or the addressed regulatory pathway [e.g. induction or downregulation (Pahnke *et al.*, 2021; Stefan and Wiese, 2019; Wiese and Stefan, 2019)]—invigorating drug design, discovery and development in general.

## Supplementary Material

btab832_Supplementary_Data
