# Supplementary material for: Structural feature-driven pattern analysis for multitarget modulator landscapes
Source: Bioinformatics. 2021 Dec 9;38(5):1385–92. doi: 10.1093/bioinformatics/btab832 (PMC8826350; doi:10.1093/bioinformatics/btab832)
Supplement: btab832_Supplementary_Data [file btab832_supplementary_data.zip › Bioinformatics_Supplementary_Figure_1_Revision_2_Final.pdf]

Structural feature-driven pattern analysis for multitarget modulator landscapes

Vigneshwaran Namasivayam[a], Katja Stefan,[b] Katja Silbermann[a], Jens Pahnke[b,c,d], Michael Wiese[a], Sven Marcel Stefan[a,b,e,*]

[a] Department of Pharmaceutical and Cellbiological Chemistry, Pharmaceutical Institute, University of Bonn, An der Immenburg 4, 53121 Bonn, Germany

[b] Department of Pathology, Section of Neuropathology, Translational Neurodegeneration Research and Neuropathology Lab (www.pahnkelab.eu), University of Oslo and Oslo University Hospital, Sognsvannsveien 20, 0372 Oslo, Norway

[c] LIED, University of Lübeck, Ratzeburger Allee 160, 23538 Lübeck, Germany

[d] Department of Pharmacology, Faculty of Medicine, University of Latvia, Jelgavas iela 1, 1004 Rīga, Latvia

[e] Cancer Drug Resistance and Stem Cell Program, University of Sydney, Kolling Building, 10 Westbourne Street, Sydney, New South Wales 2065, Australia.

* Corresponding Author: Sven Marcel Stefan (s.m.stefan@medisin.uio.no)

Phone: +47 230 71468

Supplementary Figure 1

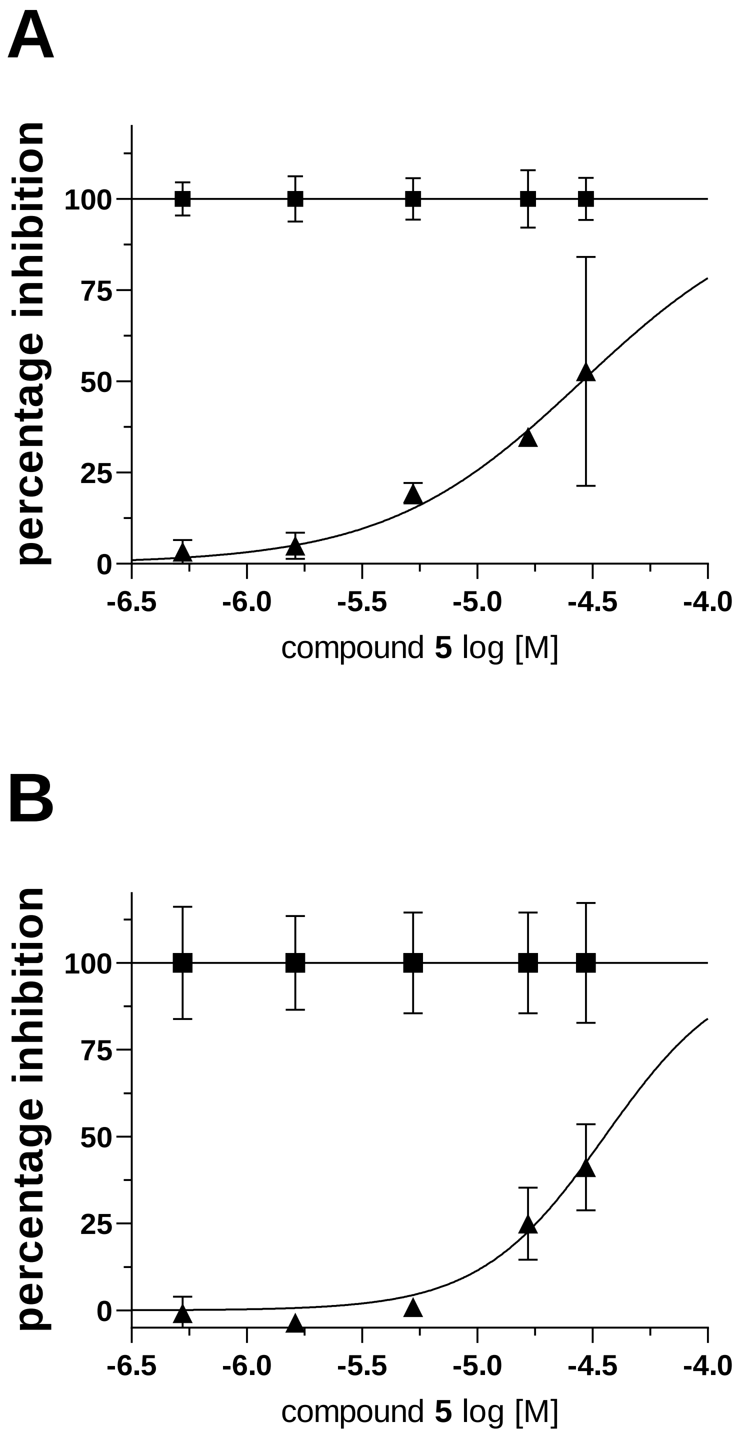

A
percentage inhibition
100
75
50
25
0
-6.5
-6.0
-5.5
-5.0
-4.5
-4.0
compound **5** log [M]
B
percentage inhibition
100
75
50
25
0
-6.5
-6.0
-5.5
-5.0
-4.5
-4.0
compound **5** log [M]

C

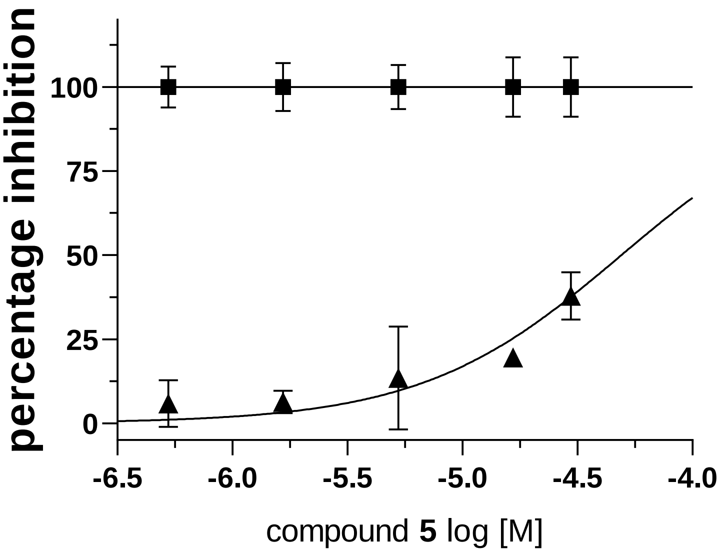

Supplementary Figure 1. Concentration-effect curves of the most potent newly discovered very weak pan-ABC transporter inhibitor, compound **5**, against ABCB1 (**A**), ABCC1 (**B**), and ABCG2 (**C**) as determined in daunorubicin (**A**–**B**) and Hoechst 33342 (**C**) assays using either ABCB1-overexpressing A2780/ADR (**A**, triangles) and sensitive A2780 (**A**, squares) cells, ABCC1-overexpressing H69AR (**B**, triangles) and sensitive H69 (**B**, squares) cells, as well as ABCG2-overexpressing MDCK II BCRP (**C**, triangles) and wild type MDCK II (**C**, squares) cells. Normalization was conducted by defining the maximal inhibition (top value) of compounds **6** (**A**), **7** (**B**), and **8** (**C**) as 100% and pure cell culture media as 0%. Shown are mean values ± SEM of three independent experiments.
