# Supplementary material for: Structural feature-driven pattern analysis for multitarget modulator landscapes
Source: Bioinformatics. 2021 Dec 9;38(5):1385–92. doi: 10.1093/bioinformatics/btab832 (PMC8826350; doi:10.1093/bioinformatics/btab832)
Supplement: btab832_Supplementary_Data [file btab832_supplementary_data.zip › Bioinformatics_Supplementary_Information_1_and_2_and_3_Revision_2_Final.pdf]

Structural feature-driven pattern analysis for multitarget modulator landscapes

Vigneshwaran Namasivayam[a], Katja Stefan[b], Katja Silbermann[a], Jens Pahnke[b,c,d], Michael Wiese[a], Sven Marcel Stefan[a,b,e,*]

[a] Department of Pharmaceutical and Cellbiological Chemistry, Pharmaceutical Institute, University of Bonn, An der Immenburg 4, 53121 Bonn, Germany

[b] Department of Pathology, Section of Neuropathology, Translational Neurodegeneration Research and Neuropathology Lab (www.pahnkelab.eu), University of Oslo and Oslo University Hospital, Sognsvannsveien 20, 0372 Oslo, Norway

[c] LIED, University of Lübeck, Ratzeburger Allee 160, 23538 Lübeck, Germany

[d] Department of Pharmacology, Faculty of Medicine, University of Latvia, Jelgavas iela 1, 1004 Rīga, Latvia

[e] Cancer Drug Resistance and Stem Cell Program, University of Sydney, Kolling Building, 10 Westbourne Street, Sydney, New South Wales 2065, Australia.

* Corresponding Author: Sven Marcel Stefan (s.m.stefan@medisin.uio.no)

Phone: +47 230 71468

Supplementary Information

Supplementary Information 1

'Putative negative substructures' were defined as the following: (i) the combined percentages of selective inhibitors of ABCB1, ABCC1, and ABCG2 of the multitarget dataset as reported earlier (Namasivayam *et al.*, 2021a) must be greater than the combined percentages of dual inhibitors of ABCB1 and ABCC1, ABCB1 and ABCG2, as well as ABCC1 and ABCG2, or (ii) the respective substructure accounted only for Class 0 compounds (inactive compounds); (iii) the percentage of Class 7 compounds must have accounted for maximally 10% of the compounds bearing the respective substructure; (iv) the respective substructure was found in at least 5 compounds in the multitarget dataset as reported earlier (Namasivayam *et al.*, 2021a); and (v) the respective substructure has not already been allocated into another multitarget substructure group. In total, 24 putative negative substructures have been identified for ongoing evaluation. Supplementary Table 1 provides their names and SMILES codes, and Supplementary Table 3 reveals their molecular formulae.

Supplementary Information 2

Before the individual contribution of each of the 103 emphasized substructures to the multitarget modulator landscape could be evaluated, the superior Class 7 and medium Class 7 compounds, the Weak and Very Weak Pan-ABC transporter inhibitors, as well as the Semi and Real Class 0 compounds have been combined and compared to each other as well as to Semi Class 7 compounds.

To evaluate the contribution of the substructures to the inner multitarget modulator landscape, the following rules applied: (i) the respective substructure must be at least 1.5-times more often present in Superior Class 7 and Medium Class 7 compounds than in Semi Class 7 compounds; (ii) the respective substructure must be at least 2.0-times more often present in Superior Class 7 and Medium Class 7 compounds than in Weak and Very Weak Pan-ABC transporter inhibitors; and (iii) the ratio between Superior Class 7 as well as Medium Class 7 compounds and Class 0 compounds must be at least 5.0. The thresholds have been chosen after visualization of the individual datasets, revealing that these thresholds efficiently divide the given data for extraction of important substructures.

To evaluate the contribution of the substructures to the outer multitarget modulator landscape, the following rules applied: (i) the respective substructure must be at least 1.5-times more often present in Weak and Very Weak Pan-ABC transporter inhibitors than in Semi Class 7 compounds; (ii) the respective substructure must be at least 2.0-times more often present in Weak and Very Weak Pan-ABC transporter inhibitors than in Superior Class 7 and Medium Class 7 compounds; and (iii) the ratio between Weak and Very Weak Pan-ABC transporter inhibitors and Class 0 compounds must be at least 1.0. This ratio has been chosen much lower in case of Weak and Very Weak Pan-ABC transporter inhibitors because the distributions of substructures followed a normal distribution. Hence, to ensure that the respective substructure peaked at Weak and Very Weak Pan-ABC transporter inhibitors, it should be at least 1.0.

However, it must be acknowledged that bioactivities do not have distinct but fluent borders, and a higher ratio would have completely abolished these types of substructures.

Supplementary Information 3

The individual substructures were evaluated and re-grouped according to the following point scheme: +++ (Superior Inner Multitarget Modulator Landscape / Superior Outer Multitarget Modulator Landscape; no rule violation); ++ (Inferior Inner Multitarget Modulator Landscape / Inferior Outer Multitarget Modulator Landscape; one rule violation); + or 0 (Intermediate Substructures; two rule violations or all rules have been violated). Supplementary Table 3 provides the 103 emphasized substructures including the 38 relevant substructures in terms of the multitarget modulator landscape of pan-ABC transporter inhibitors. **Figure 2** provides a visualization of the distribution of the Superior Inner (A), Inferior Inner (B), Superior Outer (C), as well as the Inferior Outer (D) Multitarget Modulator Landscape substructures amongst the 92 grouped focused pan-ABC transporter inhibitors as well as the 304 class 0 compounds.
