# Supplementary material for: Structural feature-driven pattern analysis for multitarget modulator landscapes
Source: Bioinformatics. 2021 Dec 9;38(5):1385–92. doi: 10.1093/bioinformatics/btab832 (PMC8826350; doi:10.1093/bioinformatics/btab832)
Supplement: btab832_Supplementary_Data [file btab832_supplementary_data.zip › Bioinformatics_Supplementary_Table_3_Revision_2_Final.pdf]

Structural feature-driven pattern analysis for multitarget modulator landscapes

Vigneshwaran Namasivayam[a], Katja Stefan,[b] Katja Silbermann[a], Jens Pahnke[b,c,d], Michael Wiese[a], Sven Marcel Stefan[a,b,e,]*

[a] Department of Pharmaceutical and Cellbiological Chemistry, Pharmaceutical Institute, University of Bonn, An der Immenburg 4, 53121 Bonn, Germany

[b] Department of Pathology, Section of Neuropathology, Translational Neurodegeneration Research and Neuropathology Lab (www.pahnkelab.eu), University of Oslo and Oslo University Hospital, Sognsvannsveien 20, 0372 Oslo, Norway

[c] LIED, University of Lübeck, Ratzeburger Allee 160, 23538 Lübeck, Germany

[d] Department of Pharmacology, Faculty of Medicine, University of Latvia, Jelgavas iela 1, 1004 Rīga, Latvia

[e] Cancer Drug Resistance and Stem Cell Program, University of Sydney, Kolling Building, 10 Westbourne Street, Sydney, New South Wales 2065, Australia.

* Corresponding Author: Sven Marcel Stefan (s.m.stefan@medisin.uio.no)

Phone: +47 230 71468

Supplementary Table 3

Supplementary Table 3. The emphasized 103 substructures in terms of the Inner and Outer Multitarget Modulator Landscape of focused pan-ABC transporter inhibitors; dark green: Superior Inner Multitarget Modulator Landscape substructures (+++; no rule violation according to Supplementary Information 2); green: Inferior Inner Multitarget Modulator Landscape substructures (++, one rule violation according to Supplementary Information 2); dark red: Superior Outer Multitarget Modulator Landscape substructures (+++; no rule violation according to Supplementary Information 2); red: Inferior Outer Multitarget Modulator Landscape substructures (++; one rule violation according to Supplementary Information 2); white: Intermediate Substructures [+ (two rule violations according to Supplementary Information 2) and 0 (all rules violated according to Supplementary Information 2)]; light grey: Tolerated Negative Substructures; dark grey: Untolerated Negative Substructures; yellow: Inconclusive Substructures; [a] identified earlier (Namasivayam *et al.* 2021a); [b] identified within the present study; [c] identified earlier (Namasivayam *et al.* 2021b);

**Substructure** (molecular formula)	**Previous Substructure Group**	**New Substructure Group**	**Contribution to Strong Pan-ABC Transporter Inhibition**	**Contribution to Weak Pan-ABC Transporter Inhibition**
4-anilinopyrimidine 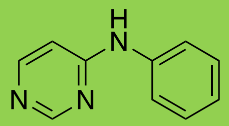	primary basic scaffolds[a]	Inferior Inner Multitarget Modulator Landscape	++	0
quinazoline 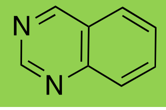	primary basic scaffolds[a]	Inferior Inner Multitarget Modulator Landscape	++	0

pyrrolo[3,2-*d*]pyrimidine 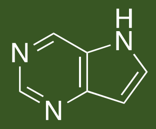	primary basic scaffolds[a]	Superior Inner Multitarget Modulator Landscape	+++	0
pyrimido[5,4-*b*]indole 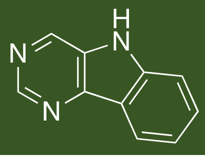	primary basic scaffolds[a]	Superior Inner Multitarget Modulator Landscape	+++	0
quinoline 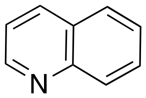	primary basic scaffolds[a]	Intermediate Substructures	+	0
thieno[2,3-*b*]pyrimidine 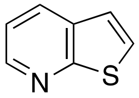	primary basic scaffolds[a]	Intermediate Substructures	+	0
1,2,4-oxadiazole 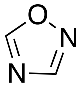	suggested basic scaffolds[a]	Intermediate Substructures	+	0
1,3,4-thiadiazole 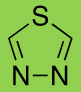	suggested basic scaffold[a]	Inferior Inner Multitarget Modulator Landscape	++	0
1,2,3,4-tetrahydroisoquinoline 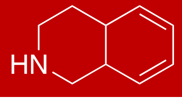	extended basic scaffold[b]	Superior Outer Multitarget Modulator Landscape	0	+++

benzochromenone 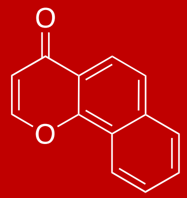	extended basic scaffold[b]	Superior Outer Multitarget Modulator Landscape	++	+++
chromenone 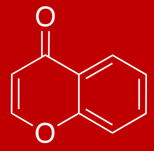	extended basic scaffold[b]	Superior Outer Multitarget Modulator Landscape	+	+++
isopropyl 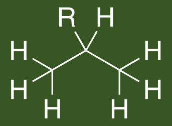	primary positive substructures[a]	Superior Inner Multitarget Modulator Landscape	+++	0
amino 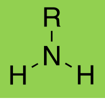	primary positive substructures[a]	Inferior Inner Multitarget Modulator Landscape	++	++
carboxylic acid ethyl ester 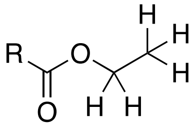	primary positive substructures[a]	Intermediate Substructures	+	0
indole 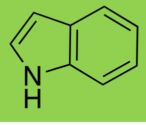	primary positive substructures[a]	Inferior Inner Multitarget Modulator Landscape	++	++

3,4,5-trimethoxyphenyl 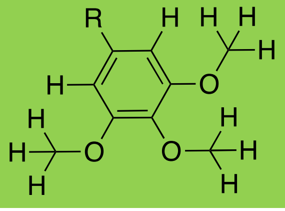	primary positive substructures[a]	Inferior Inner Multitarget Modulator Landscape	++	0
morpholine 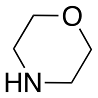	primary positive substructures[a]	Intermediate Substructures	+	0
sulfone 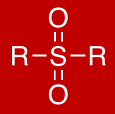	primary positive substructures[a]	Superior Outer Multitarget Modulator Landscape	0	+++
fluorine R−F	suggested positive substructures[a]	Inferior Outer Multitarget Modulator Landscape	+	++
chlorine R−Cl	suggested positive substructures[a]	Intermediate Substructures	+	+
methoxy 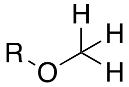	suggested positive substructures[a]	Intermediate Substructures	0	+
cyano R−CN	suggested positive substructures[a]	Superior Inner Multitarget Modulator Landscape	+++	++
piperazine 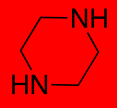	suggested positive substructures[a]	Inferior Outer Multitarget Modulator Landscape	0	++

homo-piperazine 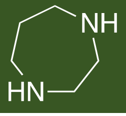	suggested positive substructures[a]	Superior Inner Multitarget Modulator Landscape	+++	+
piperidine 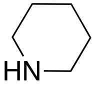	suggested positive substructures[a]	Intermediate Substructures	+	+
pyrimidine 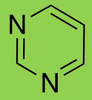	extended positive substructures[c]	Inferior Inner Multitarget Modulator Landscape	++	0
pyrrole 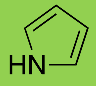	extended positive substructures[c]	Inferior Inner Multitarget Modulator Landscape	++	0
pyridine 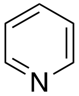	extended positive substructures[c]	Intermediate Substructures	+	0
thiophene 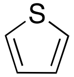	extended positive substructures[c]	Intermediate Substructures	+	0
imidazolidine 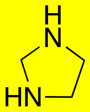	extended positive substructures[c]	Intermediate Substructures – Inconclusive Substructure	0	0
homo-piperidine 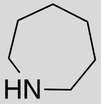	extended positive substructures[c]	Intermediate Substructures – Tolerated Negative Substructure	0	0

pyrrolidine 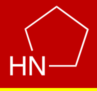	extended positive substructures[c]	Superior Outer Multitarget Modulator Landscape	0	+++
homo-morpholine 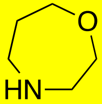	extended positive substructures[c]	Intermediate Substructures – Inconclusive Substructure	0	0
oxazolidine 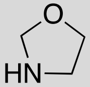	extended positive substructures[c]	Intermediate Substructures – Tolerated Negative Substructure	0	0
isoxazole 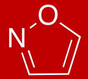	extended positive substructures[c]	Superior Outer Multitarget Modulator Landscape	0	+++
oxazole 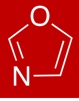	extended positive substructures[c]	Superior Outer Multitarget Modulator Landscape	0	+++
imidazole 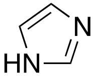	extended positive substructures[c]	Intermediate Substructures	+	0
furan 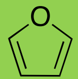	extended positive substructures[c]	Inferior Inner Multitarget Modulator Landscape	++	0
thiazole 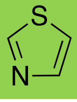	extended positive substructures[c]	Inferior Inner Multitarget Modulator Landscape	++	0

pyrazole 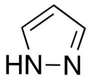	extended positive substructures[c]	Intermediate Substructures	0	0
aniline 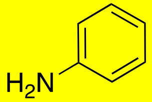	putative positive substructures[c]	Intermediate Substructures – Inconclusive Substructure	0	0
benzoyl 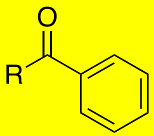	putative positive substructures[c]	Intermediate Substructures – Inconclusive Substructure	0	0
benzyl 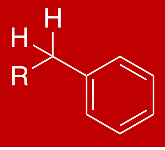	putative positive substructure[c]	Superior Outer Multitarget Modulator Landscape	+	+++
ether 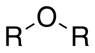	putative positive substructures[c]	Intermediate Substructures	+	+
ethylenediamine 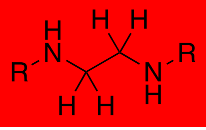	putative positive substructure[c]	Inferior Outer Multitarget Modulator Landscape	0	++
methoxyphenyl 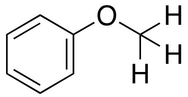	putative positive substructures[c]	Intermediate Substructures	0	+

phenol 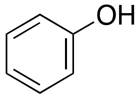	putative positive substructures[c]	Intermediate Substructures	0	+
phenyl 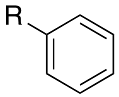	putative positive substructures[c]	Intermediate Substructures	0	+
resorcin 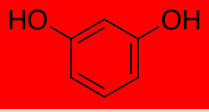	putative positive substructure[c]	Inferior Outer Multitarget Modulator Landscape	0	++
tert-butyl 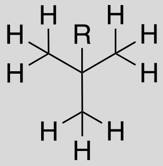	primary negative substructure[b]	Intermediate Substructures – Tolerated Negative Substructure	0	0
vinyl 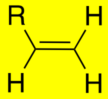	primary negative substructure[b]	Intermediate Substructures – Inconclusive Substructure	0	0
cyclopropyl 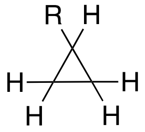	primary negative substructure[b]	Intermediate Substructures	0	0

cyclohexyl 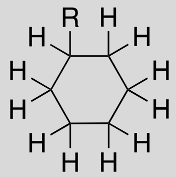	primary negative substructure[b]	Intermediate Substructures – Tolerated Negative Substructure	0	0
anellated cyclopropyl 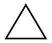	primary negative substructure[b]	Intermediate Substructures	0	0
anellated cycloheptyl 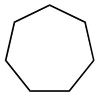	primary negative substructure[b]	Intermediate Substructures	0	0
dimethylamino 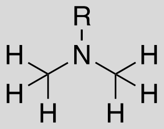	primary negative substructure[b]	Intermediate Substructures – Tolerated Negative Substructure	0	0
diethylamino 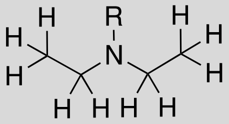	primary negative substructure[b]	Intermediate Substructures – Tolerated Negative Substructure	0	0
nitro $R-NO_2$	primary negative substructure[b]	Superior Outer Multitarget Modulator Landscape	0	+++
methylene hydroxy 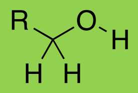	primary negative substructure[b]	Inferior Inner Multitarget Modulator Landscape	++	0
ethylene hydroxy 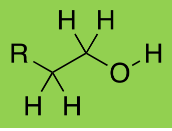	primary negative substructure[b]	Inferior Inner Multitarget Modulator Landscape	++	0

oxolane 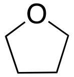	primary negative substructure[b]	Intermediate Substructures	+	0
carboxylic acid 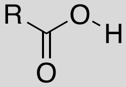	primary negative substructure[b]	Intermediate Substructures – Tolerated Negative Substructure	0	0
carboxylic acid methyl ester 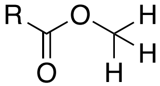	primary negative substructure[b]	Intermediate Substructures	0	0
biphenyl 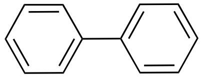	primary negative substructure[b]	Intermediate Substructures	0	0
stilbene 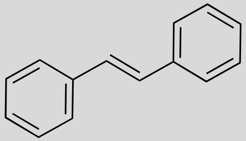	primary negative substructure[b]	Intermediate Substructures – Tolerated Negative Substructure	0	0
1,2,3-triazole 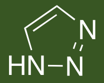	primary negative substructure[b]	Superior Inner Multitarget Modulator Landscape	+++	0
1,2,4-triazole 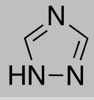	primary negative substructure[b]	Intermediate Substructures – Untolerated Negative Substructure	0	0
tetrazole 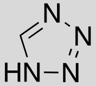	primary negative substructure[b]	Intermediate Substructures – Tolerated Negative Substructure	0	0

pyrido[2,3-*d*]pyrimidine 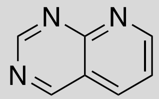	primary negative substructure[b]	Intermediate Substructures – Tolerated Negative Substructure	0	0
1,3-dihydroisobenzofuran 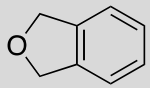	primary negative substructure[b]	Intermediate Substructures – Tolerated Negative Substructure	0	0
chalcone 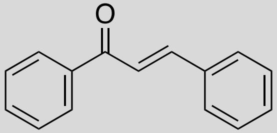	primary negative substructure[b]	Intermediate Substructures – Tolerated Negative Substructure	0	0
hydroquinone 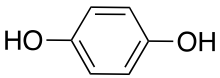	primary negative substructure[b]	Intermediate Substructures	0	0
2-methoxyphenyl 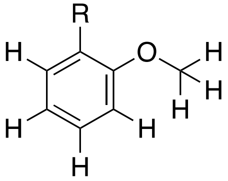	primary negative substructure[b]	Intermediate Substructures	0	0
3-methoxyphenyl 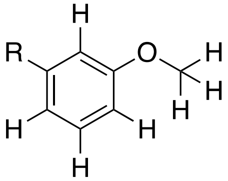	primary negative substructure[b]	Intermediate Substructures	0	0

2,5-dimethoxyphenyl 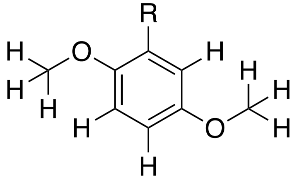	primary negative substructure[b]	Intermediate Substructures	0	0
3,5-dimethoxyphenyl 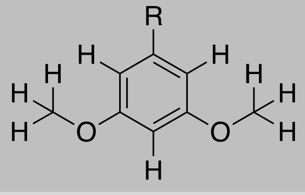	primary negative substructure[b]	Intermediate Substructures – Untolerated Negative Substructure	0	0
unsubstituted thioamide 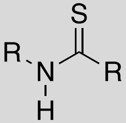	primary negative substructure[b]	Intermediate Substructures – Tolerated Negative Substructure	0	0
urea 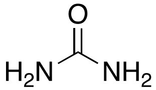	primary negative substructure[b]	Intermediate Substructures	+	0
thiourea 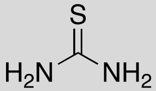	primary negative substructure[b]	Intermediate Substructures – Tolerated Negative Substructure	0	0
isoprene 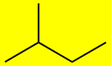	putative negative substructure[b]	Intermediate Substructures – Inconclusive Substructure	0	0
anellated cyclopentyl 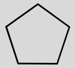	putative negative substructure[b]	Intermediate Substructures – Tolerated Negative Substructure	0	0

anellated cyclohexyl 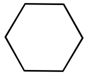	putative negative substructure[b]	Intermediate Substructures	+	0
imine 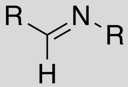	putative negative substructure[b]	Intermediate Substructures – Tolerated Negative Substructure	0	0
hydroxy 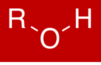	putative negative substructure[b]	Superior Outer Multitarget Modulator Landscape	0	+++
ethoxy 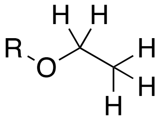	putative negative substructure[b]	Intermediate Substructures	+	0
acetyl 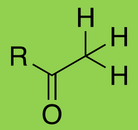	putative negative substructure[b]	Inferior Inner Multitarget Modulator Landscape	++	0
thioether 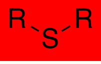	putative negative substructure[b]	Inferior Outer Multitarget Modulator Landscape	0	++
bromine R−Br	putative negative substructure[b]	Intermediate Substructures	+	0
triflumethyl 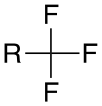	putative negative substructure[b]	Intermediate Substructures	+	+

phenethyl 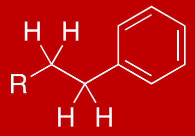	putative negative substructure[b]	Superior Outer Multitarget Modulator Landscape	0	+++
naphthalene 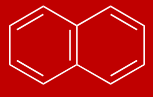	putative negative substructure[b]	Superior Outer Multitarget Modulator Landscape	+	+++
diphenylmethyl-CH 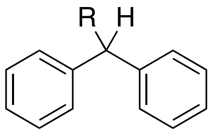	putative negative substructure[b]	Intermediate Substructures	+	0
diphenylmethyl-CRR 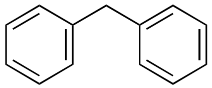	putative negative substructure[b]	Intermediate Substructures	+	0
benzo-1,3-dioxolan 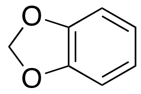	putative negative substructure[b]	Intermediate Substructures	+	0
piperonyl 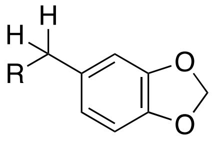	putative negative substructure[b]	Intermediate Substructures	+	0
dihydrochromene 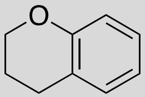	putative negative substructure[b]	Intermediate Substructures – Tolerated Negative Substructure	0	0

flavone 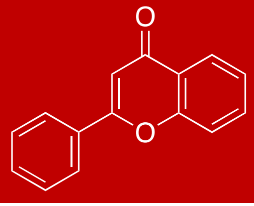	putative negative substructure[b]	Superior Outer Multitarget Modulator Landscape	+	+++
cinnamyl 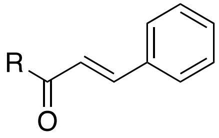	putative negative substructure[b]	Intermediate Substructures	+	0
4-methoxyphenyl 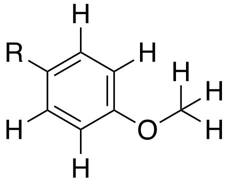	putative negative substructure[b]	Intermediate Substructures	+	0
2,4-dimethoxyphenyl 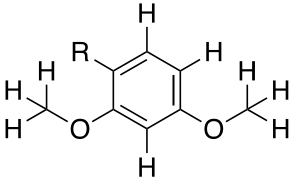	putative negative substructure[b]	Intermediate Substructures	+	0
3,4-dimethoxyphenyl 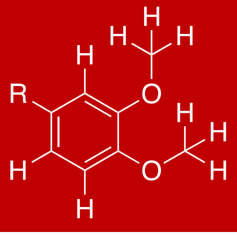	putative negative substructure[b]	Superior Outer Multitarget Modulator Landscape	0	+++

amid-H 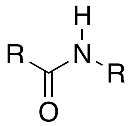	putative negative substructure[b]	Intermediate Substructures	+	+
amid-R 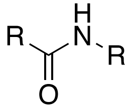	putative negative substructure[b]	Intermediate Substructures	+	+
